# Personality Traits, Strategies for Coping with Stress and the Level of Internet Addiction—A Study of Polish Secondary-School Students

**DOI:** 10.3390/ijerph15050987

**Published:** 2018-05-14

**Authors:** Joanna Chwaszcz, Bernadeta Lelonek-Kuleta, Michał Wiechetek, Iwona Niewiadomska, Agnieszka Palacz-Chrisidis

**Affiliations:** 1 Institute of Psychology, The John Paul II Catholic University of Lublin, Aleje Racławickie 14, 20-950 Lublin, Poland; wiechetek@kul.pl (M.W.); iwona.niewiadomska@kul.pl (I.N.); agnieszka.palacz@kul.pl (A.P.-C.); 2 Institute of Family Studies, The John Paul II Catholic University of Lublin, Aleje Racławickie 14, 20-950 Lublin, Poland

**Keywords:** Internet addiction, coping strategies, personality traits, young people

## Abstract

Among the many contributing factors in addictions there are also those describing the individual characteristics and ways of dealing with various life challenges. Despite numerous studies in this area, there is still no unambiguous data on the nature and specificity of this relationship in different age groups. The aim of the study was to assess the relationship between personality dimensions and strategies of coping with stress and the level of Internet addiction. The study was funded by the Ministry of Health under grant no. 93/HM/2015. The study was carried out in a group of 383 persons aged 15 to 19 (*M* = 16.6, SD = 0.77) attending secondary schools. The following research tools were used: Ten Item Personality Measure, Brief Cope and Internet Addiction Test. Both specific personality traits and styles of coping with stress are related to the addiction to the analysed medium. The personality traits most strongly associated with the risky Internet use were conscientiousness and emotional stability. An association was demonstrated between Internet addiction and the use of coping strategies, such as disengagement, substance use and self-blame. The results obtained demonstrate a major role of personality-related factors in the development of Internet addiction. The attitude to difficulties seems to be the key issue. The findings presented also make it possible to delineate the areas for improvement (e.g., through psychoeducational interventions) to protect young people from the risk of developing the addiction.

## 1. Introduction

The results of studies on the severity of Internet addiction among young people are alarming but at the same time are characterised by significant variance, which is most likely due to the diverse assessment tools and research methodologies applied, as well as to differences between study groups [1,2,3,4]. For instance, in Africa, 3.3% of young people meet the criteria for Internet addiction [5]. In China, moderate Internet addiction is found in 10.4% teenagers, while its serious levels are observed in 0.2% of the study population [6]. In Hong Kong, Internet addiction among young people ranges between 17% and 26.8% [7].

In India, it is estimated that approx. 0.7% of teenagers are addicted to the Internet [8]. The proportion of Internet addicts among Dutch teenagers is 3.7% [9]. Polish studies show that 1.3% of young people are addicted to the Internet and 12.1% are at risk of developing the addiction, i.e., in total there are 13.3% of problem Internet users [10]. The results of studies into the risk factors of pathological Internet use have shown that up to 89.9% of young people in the study group (the sample size was 11,931 female and male adolescents) display a positive correlation between pathological Internet use and repeated risky behaviours [11].

It is important to note that currently (2017), Internet addiction is yet to be recognised by the World Health Organisation or the American Psychiatric Association as an addiction or any other disease class (1992, 2013). The latest revision of the International Classification of Diseases, ICD-11, to be published in 2018, is likely to include only the associated gaming disorder [12]. However, the term has become so well-established in specialist language that it is commonly used in literature. In this paper, too, we will be using the term ‘Internet addiction’ to refer to various disorders associated with excessive use of the Internet. Excessive Internet use can be defined as the inability to control one’s Internet use, leading to psychological, social, school-related and/or professional problems [13].

Many studies on excessive Internet use or Internet addiction have confirmed the relationship between personality traits and excessive online activity. Significant correlates of Internet addiction, as identified in specialist literature, include life satisfaction [14] and self-esteem [15,16], as well as negative valence (demonstrated by demanding, needy attitudes, and eagerness to impress), and attractiveness (demonstrated by care about one’s looks, being well groomed, neat and efficient, and highly motivated) [17]. Studies conducted in 2014 in a sample of 3568 Korean Internet game players verified the risk factors for the development of Internet addiction, including: high impulsivity (odds ratio: 1.138), believed self-control (odds ratio: 1.034), anxiety (odds ratio: 1.086), pursuit of desired appetitive goals (odds ratio: 1.105), money spent on gaming (odds ratio: 1.005), weekday game time (odds ratio: 1.081), offline community meeting attendance (odds ratio: 2.060), game community membership (odds ratio: 1.393; *p* < 0.05 for all eight risk factors) [18].

A number of studies on personality-related correlates of excessive Internet use are based on the Big Five model. Meta-analyses of literature show, for instance, that there is a positive correlation between neuroticism and Internet addiction, and a negative one between the addiction and conscientiousness, agreeableness, extraversion and openness to experience in adults [19]. Multiple findings corroborate the existence of a positive correlation between neuroticism and Internet addiction [20,21,22,23,24].

A study by Montag et al., which, in addition to the Big Five personality dimensions, considered Eysenck’s Personality Inventory measures and temperament and character traits, shows that low self-directedness is a better predictor for problematic Internet use than neuroticism [25]. A negative correlation with Internet addiction, on the other hand, is shown for conscientiousness, which acts as a protective factor against problematic Internet use in this context [26,27].

A similar relationship is observed between Internet addiction and agreeableness, which, as a personality trait, can also serve as a protective factor in relation to problematic Internet use [27,28,29,30].

A negative correlation with Internet addiction has also been demonstrated for extraversion, as confirmed by various scholars [21,24,31].

The findings related to openness to experience and its relationship with Internet addiction are, in turn, conflicting. Some scholars have confirmed a negative correlation between these two variables [29,32,33]. However, there are also studies which show a positive correlation between openness to experience and Internet addiction [21,30]. Still other studies show no relationship between openness to experience and Internet addiction [28,31].

Kuss et al. argue that findings related to adults should not be extrapolated to adolescents because of the unique character of this developmental period [34]. In their study, Kuss et al. show that the factors associated with Internet addiction in young people are high neuroticism, low conscientiousness, low agreeableness, and high openness to experience, with no confirmed relationship between extraversion and the addiction [34]. In turn, in a study by Zamani et al. [35] it was high neuroticism, low conscientiousness and low extraversion that increased the risk of developing Internet addiction, while agreeableness and openness to experience did not show any such correlation.

An interesting study on the impact of the Big Five personality traits on the development of Internet addiction in young people was conducted by Zhou et al. [36]. The study explored the mediating role of teenagers’ coping styles in linking Internet addiction and personality traits. Its findings (for 998 study participants) showed that conscientiousness and agreeableness were negatively correlated with Internet addiction, while neuroticism, extraversion, and openness to experience had a positive correlation with the addiction. In-depth analyses indicated that conscientiousness indirectly influenced the development of Internet addiction in young people by reducing emotion-focused coping. Neuroticism, extraversion and openness to experience, on the other hand, indirectly influenced the development of Internet addiction in young people by strengthening emotion-focused coping. Problem-focused coping did not play any mediating role [36]. The study shows that personality traits can be correlated with Internet addiction as a result of additional mediating factors, which should be further explored by testing new Internet addiction models.

A study by Tang Jie et al. confirms the correlation between coping style and the addiction [37]. In the light of the study, a negative coping style can play a mediating role, increasing the risk of developing Internet addiction in young people who experience very stressful events in their lives. Internet addicts scored higher in negative coping, while non-addicts scored higher in positive coping [37].

A study into the mediating role of the Big Five personality traits in the development of Internet addiction in young people was also conducted by Niels van der Aa et al. [33]. Its findings indicated that daily Internet use was indirectly correlated with low well-being (i.e., loneliness, depressive mood, and low self-esteem), with compulsive Internet use having the mediating role. Moreover, daily Internet use was proven to be strongly correlated with compulsive Internet use in introverted young people with low agreeableness and emotional instability (increased neuroticism). In addition, compulsive Internet use was shown to be strongly linked to loneliness in introverted, emotionally less stable and less agreeable adolescents [38]. 

The above-mentioned examples corroborate the crucial role of the Big Five personality traits in the development of Internet addiction in both adolescents and adults. Nevertheless, it is important to note that unambiguous results are usually obtained in relation to neuroticism and conscientiousness. When it comes to the correlations between extraversion, agreeableness and openness to experience, and Internet addiction, findings are inconclusive. These differences in findings indicate that further research is needed to ascertain whether there are any such correlations. 

In this paper, we raise questions about the relationships between the Big Five personality traits and the risk of Internet addiction, and about the mediating role of coping strategies in secondary-school students. We have assumed that there is a correlation between personality dimensions and coping strategies, and Internet addiction, and that coping strategies act as mediating variables between personality and addiction.

## 2. Materials and Methods 

### 2.1. Participants

The study was conducted on a group of 383 people aged 15 to 19 (*M* = 16.60; SD = 0.77) attending Polish secondary schools of general education. The study group included 215 female and 168 male students. 30.3% of respondents lived in rural areas (*n* = 116), 14.1% in towns (*n* = 54), and 55.1% in cities (*n* = 211), while 0.5% did not disclose their place of residence (*n* = 2). Most respondents had full families (86.7%; *n* = 332), 12.8% had single-parent families (*n* = 47), and 0.5% did not disclose such information (*n* = 2). A substantial majority of respondents reported no addictions in their families (79.4%; *n* = 304). 19.3% (*n* = 74) of study participants had addicts in their families. Five persons (1.3%) did not answer this question. The most common addictions were alcohol- and nicotine-related. 

### 2.2. Procedure

The presented findings are part of a larger research project on Internet addiction operated by the Chair of Social Psychoprevention at the John Paul II Catholic University of Lublin. The study was questionnaire-based and conducted in groups during classes. Before the study was commenced, we had obtained consent from head teachers. The study was anonymous. Students were assured that their individual answers would not be disclosed to anyone. The study was conducted by trained university students participating in a psychoprevention course at the Chair of Social Psychoprevention at the Catholic University of Lublin. Prior to the survey, interviewers had introduced themselves and described the general purpose of the study. Next, they assured participants of their anonymity and distributed the questionnaires. When respondents completed the survey, interviewers collected the forms and thanked respondents for their participation. 

### 2.3. Measures

The study employed a personal information questionnaire and three research tools to measure such psychological variables as personality traits, coping strategies and the level of Internet addiction. Survey forms were printed and handed out to study participants. The forms included a general instruction and a brief description of each method used to examine key project variables. 

#### 2.3.1. Personality Traits

Personality traits were measured on the basis of the Big Five model, using the Ten Item Personality Measure [39]. This method comprises 10 items (adjectives) referring to specific individual characteristics, e.g., extraverted, enthusiastic. Respondents had to evaluate each item using a 7-point scale ranging from 1 = Disagree strongly, to 7 = Agree strongly. Each of the five personality dimensions (Extraversion, Agreeableness, Conscientiousness, Emotional Stability and Openness to Experience) was measured by two items. Scores for each dimension were averaged on the basis of the answers. Cronbach’s alphas for specific dimensions of TIPI in the Polish sample range from 0.41 to 0.67 [40]. 

#### 2.3.2. Coping Strategies

Coping was measured using the Brief Cope [41]. This tool helps determine how often the individual uses each of the 14 different strategies. When the Polish version of this tool was prepared, a factor analysis showed that these coping strategies could be grouped into four more complex classes, namely Active Coping, Helplessness, Use of Support, and Avoidance [42]. The Active Coping dimension includes a scale with the same name, i.e., Active coping, and Planning and Positive reframing scales. The Helplessness class comprises such scales as Substance use, Behavioural disengagement, and Self-blame. The Use of support is made up of such strategies as Use of emotional support and Use of instrumental support. Finally, the Avoidance dimension includes the following three strategies—Self-distraction, Denial and Venting. The remaining three strategies, i.e., Religion, Acceptance, and Humour constituted independent dimensions in the factor analysis. 

In the Brief Cope, respondents are asked to describe how they usually cope with very difficult situations. This method comprises 28 items (e.g., I’ve been turning to work or other activities to take my mind off things). Each item is evaluated by respondents using a 4-point scale, from 0 = I haven’t been doing this at all, to 3 = I’ve been doing this a lot. The score corresponds to the average answer to the statements related to the respective strategies. Each strategy is measured on the basis of two statements. Cronbach’s alphas for specific coping strategies in the Polish sample range from 0.62 to 0.89 [42].

#### 2.3.3. Internet Addiction

The level of Internet addiction was measured using The Problematic Internet Use Test by R. Poprawa [43], which is a Polish adaptation of the Internet Addiction Test by K. Young. The original version of the test includes 20 questions based on the addiction criteria specified in DSM IV. Respondents provide answers using a scale from 0 to 5, where 0 means not applicable, 1 = rarely, 2 = occasionally, 3 = frequently, 4 = often, and 5 = always. The Polish adaptation includes 3 additional items, which refer to problematic Internet use, and has 1 original item removed due to the lowest factor loading and the weakest discriminative power. Factor analysis showed that the tool had a one-factor structure with the factor score of 9.089 and 41% of the explained variance, with loadings between 0.41 and 0.72. We assumed that the test score is the sum of answers for the 22 items, which could range from 0 to 110 points. The higher the score, the greater the risk of Internet addiction. Cronbach’s alpha is .94 [44].

#### 2.3.4. Data Analysis

The data was analysed using SPSS version 24 and IBM SPSS AMOS 24 (IBM Inc., Chicago, IL, USA). Descriptive statistics were used to illustrate the distribution of scores across individual variables, i.e., personality traits, coping strategies, and Internet addiction. Next, we used Pearson’s r to evaluate the strength of the correlation between Internet addiction and personality dimensions and coping strategies. The last stage of the analysis was the development of a path model which would explain Internet addiction using personality traits and complex coping strategies. Coping was considered a mediating variable between Internet addiction and personality dimensions. 

## 3. Results

The results obtained for each tested variable are presented in Table 1. Based on Polish standards, the overall score obtained in the Internet Addiction Test for all of the interviewed secondary-school students can be considered to fall among average values [43]. A detailed analysis of the score distribution in this test shows that 1.3% (*n* = 5) of the students scored very low, 11.7% (*n* = 45) scored low, 77.8% (*n* = 298) had an average score, 8.9% (*n* = 34) scored high, and only 0.3% (*n* = 1) had a very high score. 

The assessment of personality traits in the study group shows that secondary-school students obtained relatively highest scores for openness to experience and extraversion. The lowest scores were recorded for emotional stability. The assessment of coping strategies shows that the studied secondary-school students prefer active strategies or ones which involve actions designed to use either instrumental or emotional support. They were the least likely to show behavioural disengagement, denial and substance use. 

Our analysis of the relationships between Internet addiction and the studied variables showed a number of statistically significant correlations (c.f. Table 2). These suggest that Internet addiction among Polish secondary-school students is associated with all personality variables but extraversion. Relatively strongest negative correlations were observed between the addiction and conscientiousness and emotional stability. We also demonstrated a weak correlation between Internet addiction and selected coping strategies. The attitudes to difficult situations that were found to co-occur with Internet addiction, and which could aggravate it, are Self-distraction, Venting, Behavioural disengagement, Substance use and Self-blame. On the other hand, the strategies that seem to be associated with lower risk of addiction to online media include Active coping and Planning.

**Figure 1:**
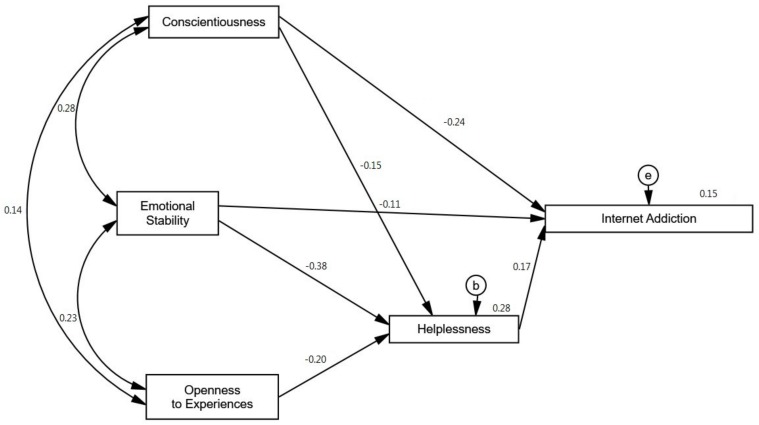
A path model explaining Internet addiction using personality traits and coping strategies. Note: e,b**—**residuals.

The final step in our analyses was to investigate the interplays between Internet addiction and personality traits and complex coping strategies. For this purpose, we used AMOS to develop a path model, in which complex coping strategies served as mediating variables between personality and Internet addiction (see Figure 1). We started from testing a saturated model in which Internet addiction was a dependent variable; specific personality traits (Extraversion, Agreeableness, Conscientiousness, Emotional stability, Openness to experience) were independent variables and complex coping strategies (Active coping, Helplessness, Use of support, Avoidance) were mediators. Then, insignificant paths were removed. A model so modified has satisfactory fit indices (RMSEA < 0.001; χ^2^(1) = 0.001; *p* = 0.99). In our model, the variables that best explained the Internet addiction level were conscientiousness, emotional stability, and openness to experience, and helplessness as a complex coping strategy. These variables accounted for 15% of the variance in Internet addiction. All the paths included in the model proved statistically significant. Low conscientiousness (β = −0.24) can directly contribute to the development of the addiction. In the case of openness to experience, this correlation is mediated by helplessness. As regards emotional stability, its impact on Internet addiction is both direct (β = −0.11) and mediated by helplessness. Low emotional stability contributes to problem Internet use, but can also make the individual more helpless, which, in turn, increases the risk of Internet addiction. It is worth emphasising that Helplessness is a partial mediator between Internet Addiction and Conscientiousness just as it is between Internet Addiction and Emotional stability. On the other hand, Helplessness is a full mediator between openness to experience and problematic Internet use. Detailed information about the overall, direct and indirect effect of analysed variables computed by bias-corrected percentile methods is reported in Table 2. It can be concluded from the analysis of the indirect to overall effect ratio that the indirect effect fully explains the overall effect of openness to experience. For emotional stability and conscientiousness, however, the ratios are relatively low at 0.41 and 0.12, respectively. This result suggests that research is needed to identify yet further mediators between personality and Internet addiction.

## 4. Discussion

Our findings are consistent with those of other scholars in relation to personality risk factors of Internet addiction. 

The positive correlation between Internet addiction and emotional instability is confirmed by studies in both adults and adolescents [9,19,20,21,22,23,24,33,36].

A negative relationship between the risk of Internet addiction and conscientiousness was observed in studies by Montag et al., 2011 [26]; Zamani, 2011 [35] and Randler et al., 2014 [27]. A negative correlation between Internet addiction and agreeableness was found by Andreassen et al., 2013 [28]; Durak, Senol-Durak, 2014 [29]; Hwang et al., 2014 [30]; Randler et al., 2014 [27], Kuss et al., 2013 [9], and Zhou, 2017 [36]. 

Another trait associated with Internet addiction in our study in secondary-school students was openness to experience, which showed a negative correlation with the risk of the addiction. This was also confirmed by Durak, Senol-Durak, 2014 [29]; Servidio, 2014 [32]; Van der Aa et al., 2009 [33], and Kuss et al., 2013 [9].

Our study did not reveal any relationship between the risk of developing the addiction and extraversion. This is consistent with the findings reported by Kuss et al., 2013 [9]. A negative relationship between extraversion and Internet addiction was demonstrated by Buckner et al., 2012 [31]; Rahmani, Lavasani, 2011 [21]; Zamani, 2011 [35]; Yan, Li, Sui, 2014 [24], and Kayiş et al., 2016 [19].

In addition, our study showed a clear relationship between negative coping strategies and the risk of Internet addiction. The risk of developing the addiction is associated with the teenagers’ use of such strategies as distraction, cessation of activities leading to problem resolution, release of emotions, self-blame, and substance use. Similar findings were reported by Tang Jie et al. [37], who suggested a relationship between negative coping styles and an increased risk of media addiction. Persons with developed Internet addiction display emotional coping strategies, which can additionally strengthen any depressive and autoaggressive tendencies in these individuals [38].

Zhou [36] observed an important role of emotion-focused coping style, which, when co-occurring with neuroticism, increased the risk of Internet addiction. Our study found a relationship between emotional instability and the risk of Internet addiction, which is reinforced when young people use emotion-focused strategies described as helplessness, namely substance use, behavioural disengagement and self-blame. In addition, the relationship between openness to experience and risk of Internet addiction was found to be mediated by helplessness.

### Limitations

The research was conducted in one voivodeship, which is one of the poorest in Poland. It is worth to considerate to replicate it in other regions of the country. There is also a limitation in form of short study method to asses personality traits which has relatively low measures of reliability. It appears to be important to conduct similar research with application of more reliable methods. The last limitation of the research is the study group—high school students. It is worth to conduct the research on other age groups to asses whether noted results are similar.

## 5. Conclusions

To summarise, the study explored possible correlations between the Big Five personality traits and the risk of Internet addiction in young people and sought mediating variables for these correlations. Our findings are consistent with those of other scholars in relation to the correlations found between four out of five personality factors and the risk of Internet addiction. These correlations are observed between emotional stability, conscientiousness, agreeableness and openness to experience, and the risk of Internet addiction. Also, the correlations between Internet addiction and such traits as openness to experience and emotional stability are mediated by a factor referred to as helplessness. In our path model, no direct or indirect correlation was found between extraversion and the risk of Internet addiction. In correlation analyses, in turn, positive relationships were observed for such coping strategies as self-distraction, behavioural disengagement, venting, and self-blame, and the risk of developing the addiction, while negative correlations were established between active coping and planning, and the risk of Internet addiction.

A practical conclusion offered by the study is that some personality traits can act as protective factors, while others can serve as risk factors, in the context of Internet addiction. These are emotional stability, conscientiousness, agreeableness, and openness to experience. The strongest correlations are found between emotional stability and conscientiousness, and the risk of developing the addiction. Emotional stability and openness to experience, on the other hand, show a relationship between the risk of Internet addiction, which can be direct or mediated by helplessness-related strategies. The coping strategies which negatively correlate with Internet addiction are active coping and planning. A risk factor is the use of nonconstructive helplessness-related coping strategies, such as behavioural disengagement, substance use, self-blame, and venting. 

As regards the correlation between personality traits and the risk of Internet addiction, it seems reasonable to argue that, given their basis, personality traits can be considered as contributing to the risk of addiction. Coping strategies can be both a cause and a consequence of excessive involvement in online activity. An interesting observation made in our study was that helplessness, defined as behavioural disengagement, substance use and self-blame, acted as a mediator of the relationship between emotional instability and openness to experience, and the risk of Internet addiction. Low emotional stability is associated with increased use of helplessness-related strategies, which, in turn, increases the risk of developing the addiction. Similarly, low openness to experience is associated with helplessness-related strategies, which increases the risk of Internet addiction. Given the constancy of the Big Five personality traits, and the fact that they are to a large extent inherited, prevention measures are not designed to induce a change in personality but to develop constructive and active coping strategies to minimise the risk of Internet addiction. Such measures are particularly important during adolescence, when children are already aware of the reasons for, and consequences of, their behaviour over time, but are still socially malleable, having no fixed cognitive or behavioural patterns which are characteristic of an addiction. 

The exploration of the correlations between personality traits and the risk of addiction in practical terms is justified only when it addresses questions about the factors which mediate these correlations and can be changed in the process of socialisation. Our study has provided only a partial explanation of the reality, which must be explored further.

## Figures and Tables

**Table 1:** Interplay between Internet addiction, personality traits and coping strategies (Pearson’s correlation coefficient).

Variables	1. Internet Addiction	2.	3.	4.	5.	6.	7.	8.	9.	10.	11.	12.	13.	14.	15.	16.	17.	18.	19.	20.	21.	22.	23.
Personality traits																							
2. Extraversion	−0.08																						
3. Agreeableness	−0.12 *	0.03																					
4. Conscientiousness	−0.32 **	0.01	0.22 **																				
5. Emotional stability	−0.26 **	0.08	0.24 **	0.28 **																			
6. Openness to experience	−0.11 *	0.35 **	0.09	0.14 **	0.23 **																		
Coping strategies																							
7. Active coping	−0.19 **	0.21 **	0.14 **	0.30 **	0.28 **	0.29 **																	
8. Planning	−0.13 **	0.16 **	0.05	0.25 **	0.18 **	0.18 **	0.59 **																
9. Positive reframing	−0.04	0.13 **	0.15 **	0.10 *	0.24 **	0.23 **	0.30 **	0.36 **															
10. Acceptance	0.06	0.03	0.06	−0.01	0.04	0.10	0.21 **	0.25 **	0.33 **														
11. Humour	0.07	0.04	−0.05	−0.21 **	−0.07	0.09	−0.08	−0.04	0.28 **	0.17 **													
12. Religion	−0.01	−0.02	0.06	0.11 *	−0.01	0.05	0.12 *	0.19 **	0.21 **	0.10	−0.01												
13. Use of emotional support	−0.05	0.29 **	0.08	0.08	−0.01	0.16 **	0.26 **	0.16 **	0.14 **	0.02	−0.01	0.20 **											
14. Use of instrumental support	−0.01	0.23 **	0.11 *	0.08	−0.08	0.08	0.18 **	0.18 **	0.12 *	0.00	−0.06	0.22 **	0.78 **										
15. Self-distraction	0.12 *	−0.06	−0.05	−0.03	−0.16 **	−0.12 *	−0.14 **	−.05	0.13 *	0.11 *	0.18 **	0.12 *	0.10	0.12 *									
16. Denial	0.08	−0.02	−0.05	−0.08	−0.20 **	−0.08	−0.14 **	−0.10	−0.03	−0.10 *	0.21 **	0.04	0.02	0.05	0.16 **								
17. Venting	0.11 *	0.07	−0.10	−0.17 **	−0.35 **	−0.03	−0.08	0.01	0.12 *	0.03	0.17 **	0.05	0.26 **	0.28 **	0.25 **	0.21 **							
18. Substance use	0.13 *	0.09	−0.18 **	−0.16 **	−0.20 **	−0.02	−0.09	−0.04	0.02	−.01	0.21 **	−0.10	0.08	0.06	0.04	0.35 **	0.16 **						
19. Behavioural disengagement	0.29 **	−0.23 **	−0.08	−0.29 **	−0.30 **	−0.34 **	−0.43 **	−0.30 **	−0.19 **	−0.10 *	0.18 **	−0.10	−0.13 *	−0.06	0.14 **	0.36 **	0.15 **	0.31 **					
20. Self-blame	0.20 **	−0.11 *	−0.15 **	−0.16 **	−0.47 **	−0.27 **	−0.12 *	0.03	−0.13 *	0.03	0.07	0.02	−0.08	−0.03	0.19 **	0.22 **	0.26 **	0.09	0.31 **				
Complex coping strategies																							
21. Active coping	−0.15 **	0.21 **	0.15 **	0.27 **	0.29 **	0.29 **	0.78 **	0.83 **	0.73 **	0.34 **	0.08	0.22 **	0.24 **	0.20 **	−0.01	−0.11 *	0.03	−.04	−0.38 **	−0.09			
22. Helplessness	0.29 **	−0.13 *	−0.19 **	−0.29 **	−0.47 **	−0.31 **	−0.30 **	−0.14 **	−0.13 **	−0.04	0.21 **	−0.08	−0.06	−0.02	0.18 **	0.44 **	0.28 **	0.64 **	0.76 **	0.71 **	−0.24 **		
23. Use of support	−0.03	0.28 **	0.10	0.09	−0.04	0.13 *	0.23 **	0.18 **	0.14 **	0.01	−0.04	0.22 **	0.95 **	0.94 **	0.12 *	0.04	0.29 **	0.07	−0.10 *	−0.05	0.23 **	−0.05	
24. Avoidance	0.15 **	0.01	−0.09	−0.14 **	−0.34 **	−0.11 *	−0.17 **	−0.07	0.10 *	0.02	0.27 **	0.11 *	0.19 **	0.22 **	0.68 **	0.68 **	0.71 **	0.26 **	0.31 **	0.33 **	−0.05	0.43 **	0.21 **
*M*	27.73	4.86	4.78	**4.46**	**4.02**	**5.41**	**2.11**	**2.01**	**1.57**	**1.87**	**1.18**	**1.07**	**1.77**	**1.75**	**1.63**	**0.70**	**1.49**	**0.42**	**0.74**	**1.60**	**1.90**	**0.92**	**1.76**
*SD*	15.43	1.53	1.24	**1.54**	**1.60**	**1.29**	**.66**	**0.75**	**0.80**	**0.70**	**0.79**	**0.94**	**0.88**	**0.85**	**0.72**	**0.75**	**0.73**	**0.73**	**0.74**	**0.86**	**0.57**	**0.55**	**0.82**

* *p* ≤ 0.05; ** *p* ≤ 0.01.

**Table 2:** Overall, direct and indirect effect of personality traits on Internet Addiction mediated by Hopelessness.

Type of Effect:	Openness to Experience	Emotional Stability	Conscientiousness
Overall effect	−0.04 **	−0.17 **	−0.26 **
Direct effect	-	−0.11	−0.24 **
Indirect effect	−0.04 **	−0.07 **	−0.03 *

* *p* < 0.05; ** *p* < 0.01.
